# PCR-RFLP analyses of *Leishmania* species causing cutaneous and mucocutaneous leishmaniasis revealed distribution of genetically complex strains with hybrid and mito-nuclear discordance in Ecuador

**DOI:** 10.1371/journal.pntd.0007403

**Published:** 2019-05-06

**Authors:** Hirotomo Kato, Eduardo A. Gomez, Chisato Seki, Hayato Furumoto, Luiggi Martini-Robles, Jenny Muzzio, Manuel Calvopiña, Lenin Velez, Makoto Kubo, Ahmed Tabbabi, Daisuke S. Yamamoto, Yoshihisa Hashiguchi

**Affiliations:** 1 Division of Medical Zoology, Department of Infection and Immunity, Jichi Medical University, Tochigi, Japan; 2 Departamento de Parasitologia y Medicina Tropical, Facultad de Ciencias Medicas, Universidad Catolica de Santiago de Guayaquil, Guayaquil, Ecuador; 3 Laboratory of Parasitology, Department of Disease Control, Graduate School of Veterinary Medicine, Hokkaido University, Hokkaido, Japan; 4 Hospital de Especialidades Guayaquil “Dr. Abel Gilberto Pinton”, Ministerio de Salud Publica, Guayaquil, Ecuador; 5 Departamento de Parasitologia, Insitituto de Investigacion de Salud Publica, Guayaquil, Ecuador; 6 Escuela de Medicina, Facultad de Ciencias de la Salud, Universidad De Las Américas (UDLA), Quito, Ecuador; 7 Division of Immunology, Kitasato University School of Allied Health Sciences, Kanagawa, Japan; Centro de Pesquisa Gonçalo Moniz-FIOCRUZ/BA, BRAZIL

## Abstract

PCR-Restriction Fragment Length Polymorphism (RFLP) analyses targeting multiple nuclear genes were established for the simple and practical identification of *Leishmania* species without using expensive equipment. This method was applied to 92 clinical samples collected at 33 sites in 14 provinces of Ecuador, which have been identified at the species level by the kinetoplast cytochrome *b* (*cyt* b) gene sequence analysis, and the results obtained by the two analyses were compared. Although most results corresponded between the two analyses, PCR-RFLP analyses revealed distribution of hybrid strains between *Leishmania (Viannia) guyanensis* and *L*. *(V*.*) braziliensis* and between *L*. *(V*.*) guyanensis* and *L*. *(V*.*) panamensis*, of which the latter was firstly identified in Ecuador. Moreover, unexpected parasite strains having the kinetoplast *cyt* b gene of *L*. *(V*.*) braziliensis* and nuclear genes of *L*. *(V*.*) guyanensis*, *L*. *(V*.*) panamensis*, or a hybrid between *L*. *(V*.*) guyanensis* and *L*. *(V*.*) panamensis* were identified. This is the first report of the distribution of a protozoan parasite having mismatches between kinetoplast and nuclear genes, known as mito-nuclear discordance. The result demonstrated that genetically complex *Leishmania* strains are present in Ecuador. Since genetic exchanges such as hybrid formation were suggested to cause higher pathogenicity in *Leishmania* and may be transmitted by more species of sand flies, further country-wide epidemiological studies on clinical symptoms, as well as transmissible vectors, will be necessary.

## Author summary

Leishmaniasis caused by intracellular protozoa of the genus *Leishmania* is a neglected tropical disease widely distributing worldwide, especially in tropical and subtropical areas. Approximately 20 species are known to be pathogenic to humans, of which eight species have been recorded as causative agents of cutaneous and mucocutaneous leishmaniases in Ecuador. Since infecting species are the major determinant of clinical outcomes, identification at the species level is important for the treatment and prognosis. The parasite species have been identified conventionally by multilocus enzyme electrophoresis (MLEE) and recently by genetic analysis such as sequencing and genotyping. In the present study, PCR-Restriction Fragment Length Polymorphism (RFLP) targeting multiple nuclear genes was employed, and the results were compared with those obtained by kinetoplast cytochrome *b* (*cyt* b) gene sequence analysis, which is widely applied to species identification. Although most results corresponded between the two analyses, PCR-RFLP revealed presence of unexpected genetically complex *Leishmania* strains having characteristics of hybrid and mito-nuclear discordance. Since hybrid strains of *Leishmania* were suggested to increase disease severity and may be transmitted by a wider range of sand fly species, careful epidemiological research, including clinical courses and vector research, will be needed.

## Introduction

Leishmaniasis, caused by protozoan parasites of the genus *Leishmania*, is a neglected tropical disease widely distributed worldwide, especially in tropical and subtropical areas, affecting at least 12 million people in 96 countries [1]. Approximately 20 *Leishmania* species belonging to the subgenera *Leishmania* (*Leishmania*), *Leishmania* (*Viannia*) and *Leishmania (Mundinia)* are pathogenic to humans [1, 2]. Since infected parasite species is known to be the major determinant of clinical outcomes in leishmaniasis [1], identification of the causative parasite is important for appropriate treatment and prognosis.

*Leishmania* species have been classified conventionally by multilocus enzyme electrophoresis (MLEE) [3, 4]. Genetic analysis of kinetoplast and nuclear targets, such as cytochrome *b* (*cyt* b), cysteine protease (*cpb*), heat shock protein 70 (*hsp70*) genes and the internal transcribed spacer (ITS) regions of ribosomal RNA, has commonly been used for species identification due to its sensitivity, simplicity and reliability [5–13]. In addition, a simple PCR-Restriction Fragment Length Polymorphism (RFLP), which does not require costly equipment, was developed for species identification, and the ITS region and *hsp70* gene are widely applied to epidemiological studies [11, 14–19].

In Ecuador, leishmaniasis is endemic in Pacific coast, Andean highland, and Amazonian areas, and eight species, *Leishmania* (*Leishmania*) *mexicana*, *L*. *(L*.*) amazonensis*, *L*. *(L*.*) major*-like, *L*. *(Viannia) guyanensis*, *L*. *(V*.*) panamensis*, *L*. *(V*.*) braziliensis*, *L*. *(V*.*) naiffi*, and *L*. *(V*.*) lainsoni*, have been recorded as causative agents of cutaneous leishmaniasis (CL) and mucocutaneous leishmaniasis (MCL) [8, 20, 21]. Of these, distribution of *L*. *(L*.*) amazonensis* and *L*. *(L*.*) major*-like have been reported to be localized, and infections by them have not been reported recently [8, 21]. Infection by *L*. *(V*.*) guyanensis* together with its closely-related species, *L*. *(V*.*) panamensis*, has been identified from CL patients in Pacific coast areas by MLEE [21–24]; however, our recent *cyt* b gene analysis revealed a wide range distribution of *L*. *(V*.*) guyanensis*, without detecting any *L*. *(V*.*) panamensis* in these areas [8]. These results suggest that endemic species may change, or the reported results may be caused by the discordance between the MLEE analysis and kinetoplast *cyt* b gene analysis employed for species identification. Recently, a countrywide epidemiological study was carried out based on the *cyt* b sequence analysis and it identified *L*. *(V*.*) guyanensis* and *L*. *(V*.*) braziliensis* widely in Pacific coast and Amazonian areas and *L*. *(L*.*) mexicana* in Andean high lands as current major causative species in Ecuador [8]. Additionally, *L*. *(V*.*) naiffi* and *L*. *(V*.*) lainsoni* were recently recorded in Amazonian areas [8, 20, 25].

In this study, a simple and practical method for the identification of *Leishmania* species in Ecuador was established on the basis of PCR-RFLP analyses targeting mannose phosphate isomerase (*mpi*) and 6-phosphogluconate dehydrogenase (*6pgd*) genes, and the result was compared with that obtained by the *cyt* b gene sequence analysis. This study demonstrated the presence of genetically complex *Leishmania* strains in Ecuador, and strongly suggested the importance of applying multiple target approaches to enhance the reliability of species identification and to characterize more detailed genetic properties of the parasite.

## Methods

### Parasite specimens and clinical samples

Frozen stocks of 24 parasite strains of five *Leishmania* species [*L*. *(V*.*) guyanensis*, *L*. *(V*.*) panamensis*, *L*. *(V*.*) braziliensis*, *L*. *(L*.*) major*-like, *L*. *(L*.*) mexicana*] that were isolated from CL patients in Ecuador and identified at the species level by MLEE [22–24] (Table 1) were spotted on an FTA Classic Card (Whatman, Newton Center, MA) and subjected to sequence analysis. Three strains of *L*. *(V*.*) naiffi* identified by *cyt* b gene analysis [25, 26] were also utilized (Table 1).

**Table 1:** *Leishmania* strains isolated in Ecuador.

**Species**	**Strains**
*L*. *(V*.*) guyanensis*	MHOM/EC/05/EC4
*L*. *(V*.*) guyanensis*	MHOM/EC/05/EC6
*L*. *(V*.*) guyanensis*	MHOM/EC/05/EC7
*L*. *(V*.*) guyanensis*	MHOM/EC/05/EC8
*L*. *(V*.*) guyanensis*	MHOM/EC/05/EC9
*L*. *(V*.*) guyanensis*	MHOM/EC/05/EC11
*L*. *(V*.*) guyanensis*	MHOM/EC/05/EC12
*L*. *(V*.*) guyanensis*	MHOM/EC/05/XPEA1
*L*. *(V*.*) guyanensis*	MHOM/EC/05/LM3
*L*. *(V*.*) panamensis*	MHOM/EC/87/G05
*L*. *(V*.*) panamensis*	MHOM/EC/87/G06
*L*. *(V*.*) panamensis*	MHOM/EC/87/G07
*L*. *(V*.*) panamensis*	MHOM/EC/88/INH23
*L*. *(V*.*) braziliensis*	MHOM/EC/00/Ppa20
*L*. *(V*.*) braziliensis*	MHOM/EC/00/LASU22
*L*. *(V*.*) naiffi*	07tor
*L*. *(V*.*) naiffi*	13tor1
*L*. *(V*.*) naiffi*	13tor2
*L*. *(L*.*) major*-like	MHOM/EC/87/G09
*L*. *(L*.*) major*-like	MHOM/EC/88/PT115
*L*. *(L*.*) mexicana*	MHOM/EC/88/PT23
*L*. *(L*.*) mexicana*	MHOM/EC/88/PT27
*L*. *(L*.*) mexicana*	MHOM/EC/88/PT29
*L*. *(L*.*) mexicana*	MHOM/EC/88/PT103
*L*. *(L*.*) mexicana*	MHOM/EC/92/HU3
*L*. *(L*.*) mexicana*	MHOM/EC/92/HU4
*L*. *(L*.*) mexicana*	MHOM/EC/00/HU6

Most of the clinical samples employed in this study were collected from patients suspected of CL in the previous study [8, 20], and each 3 samples newly obtained from Provinces of Manabi and Santo Domingo de los Tsachilas, all of which were identified as *L*. *(V*.*) guyanensis* by the *cyt* b gene analysis, were included in this study. *Leishmania* parasites were identified on the basis of *cyt* b sequence analysis [8, 20]. The samples were collected at 33 sites in 14 provinces of Ecuador (S1 Fig). Residual tissue materials were spotted onto an FTA Classic Card, after taking scraped margin samples of active lesions for routine diagnosis. Two-mm-diameter disks of FTA card were punched out from each filter paper, washed three times with an FTA Purification Reagent (Whatman), and subjected to PCR amplification.

### PCR and sequence analysis

PCR primers for amplification of *cyt* b, *hsp70*, mannose phosphate isomerase (*mpi*) and 6-phosphogluconate dehydrogenase (*6pgd*) gene fragments were designed based on the sequence regions conserved among species (Table 2). PCR amplification with a pair of outer primers was performed with 30 cycles of denaturation (95°C, 1 min), annealing (55°C, 1 min) and polymerization (72°C, 2 min) using Ampdirect Plus reagent (Shimadzu Biotech, Tsukuba, Japan). Each 0.5-μl portion of the PCR product was reamplified with inner primers under the same condition described above. The products were cloned into the pGEM-T Easy Vector System (Promega, Madison, WI) and sequences were determined on both strands by the dideoxy chain termination method using a BigDye Terminator v3.1 Cycle Sequencing Kit (Applied Biosystems, Foster City, CA). Primers for amplification of a partial sequence of the kinetoplast cytochrome oxidase subunit II-NADH dehydrogenase subunit I region (COII-ND1) were also designed based on the sequences conserved among species (Table 2). The COII-ND1 sequences were determined on both strands by direct sequencing with inner primers, L.COII-2S and L.COII-2R. Restriction enzyme mapping was performed *in silico* by using BioEdit Sequence Alignment Editor to obtain species-specific RFLP patterns.

**Table 2:** Primer sequences used in this study.

**Target gene**	**Primer**		**Primer sequence (5’ to 3’)**	**Expected** **amplicon size (bp)**
cytochrome *b*	outer	L.cyt-AS	GCGGAGAGRARGAAAAGGC	978
(*cyt* b)		L.cyt-AR	CCACTCATAAATATACTATA	
	inner	L.cyt-S	GGTGTAGGTTTTAGTYTAGG	866
		L.cyt-R	CTACAATAAACAAATCATAATATRCAATT	
cytochrome oxidase	outer	L.COII-1S	AACATAGTTCTCATTGCAGA	954
subunit II—NADH		L.COII-1R	ACAMCGRCCAGGTTCTCTAC	
dehydrogenase subunit 1	inner	L.COII-2S	AATGCAACATGCAGTTATWA	736
(COII-ND1)		L.COII-2R	AATGAATGTATAACATCAAC	
heat shock protein 70	outer	L.HSP-OS	GGGCACGACGTACTCGTGCG	1,931
(*hsp70*)		L.HSP-OR	AGTCGACCTCCTCGACCTTG	
	inner	L.HSP-IS2	CCGTCGTACGTTGCGTTCAC	1,735
		L.HSP-IR2	TGCTCTGGTACATCTTGGTC	
	outer*	L.HSP-Ty1S	GGCGAGCGCGCGATGACGAA	847
		L.HSP-OR	AGTCGACCTCCTCGACCTTG	
	inner*	L.HSP-Ty2S	CGTTCGACTTGTCCGGCATC	468
		L.HSP-IR2	TGCTCTGGTACATCTTGGTC	
mannose phosphate	outer	L.MPI-OS2	GCCTGGGGCAAGRATGCCGC	1,214
isomerase		L.MPI-OR	CTCAAGTCGTTGGTCGACGC	
(*mpi*)	inner	L.MPI-IS2	CGTCCAGCTTCGTGGCRAAG	1,130
		L.MPI-IR2	GCCGTACGGYACCGCAAAGC	
6-phosphogluconate	outer	L.6PGD-OS	GAACGACCTCGGYATTATCG	1,346
dehydrogenase		L.6PGD-OR	GACACCAGCTGTCCGTACGG	
(*6pgd*)	inner	L.6PGD-IS	GCCCTGAACATCGCCGAGAA	1,272
		L.6PGD-IR	CGTGTACATGGCGTTGATGT	

*The primer sets were used for the PCR-RFLP analysis to differentiate *L*. *(V*.*) guyanensis* from *L*. *(V*.*) panamensis*.

### PCR-Restriction Fragment Length Polymorphism (RFLP) analysis

Clinical samples spotted on FTA cards, in which parasites were identified by *cyt* b gene analysis in a previous study, were subjected to PCR-RFLP analysis. PCR amplifications targeting *mpi* and *6pgd* were performed as described above using a high fidelity DNA polymerase, KOD plus (Toyobo, Osaka, Japan). The PCR products were digested by restriction enzymes *Hae*III, *Hap*I, and *Bst*XI for the *mpi* gene and *Bsp*1286I and *Hin*fI for the *6pgd* gene, and resulting restriction fragment patterns were analyzed by 2% agarose gel electrophoresis. GeneRuler 100 bp Plus DNA Ladder (Thermo Fisher Scientific, Waltham, MA) was used as a DNA size marker. The gel was stained with GelRed Nucleic Acid Gel Stain (Biotium, Hayward, CA), and DNA fragments were visualized with UV transilluminator.

Differentiation between *L*. *(V*.*) guyanensis* and *L*. *(V*.*) panamensis* was performed by restriction enzyme-digestion of the *hsp*70 gene fragment [27]. Briefly, the *hsp*70 gene fragment was amplified by a nested PCR using sets of outer primers (L.HSP-Ty1S and L.HSP-OR) and inner primers (L.HSP-Ty2S and L.HSP-IR2) (Table 2). The amplicons were digested with a restriction enzyme, *Bcc*I, and resulting fragment patterns were analyzed by 3% agarose gel electrophoresis.

### Ethics statement

Clinical samples were collected by local physicians and well-trained laboratory technicians of health centers of the Ministry of Health, Ecuador. For routine parasitological diagnosis, scratching smear samples of skin lesions were taken from suspected leishmaniasis patients at health centers. In this study, only residual tissue materials were collected after the routine procedure to minimize the burden on patients. Signed consent was obtained from the adult subjects and from the children’s parents or guardians, prior to the diagnostic procedures at each health center of the Ministry, providing information on the process of diagnosis and *Leishmania* species analysis, following the guidelines of the Ethics Committee of the Ministry. The subjects studied were volunteers in routine diagnosis/screening and treatment programs promoted by the Ministry. All routine laboratory examinations were carried out free of charge, and treatment with specific drug, meglumine antimoniate (Glucantime) was also offered free of charge at each health center. The study was approved by the ethics committee of the Graduate School of Veterinary Medicine, Hokkaido University (approval number: vet26-4) and Jichi Medical University (approval number: 17–080) [8].

## Results

### Sequence analysis of *cyt b*, *hsp70*, *mpi* and *6pgd* genes from *Leishmania* strains

**Fig 1:**
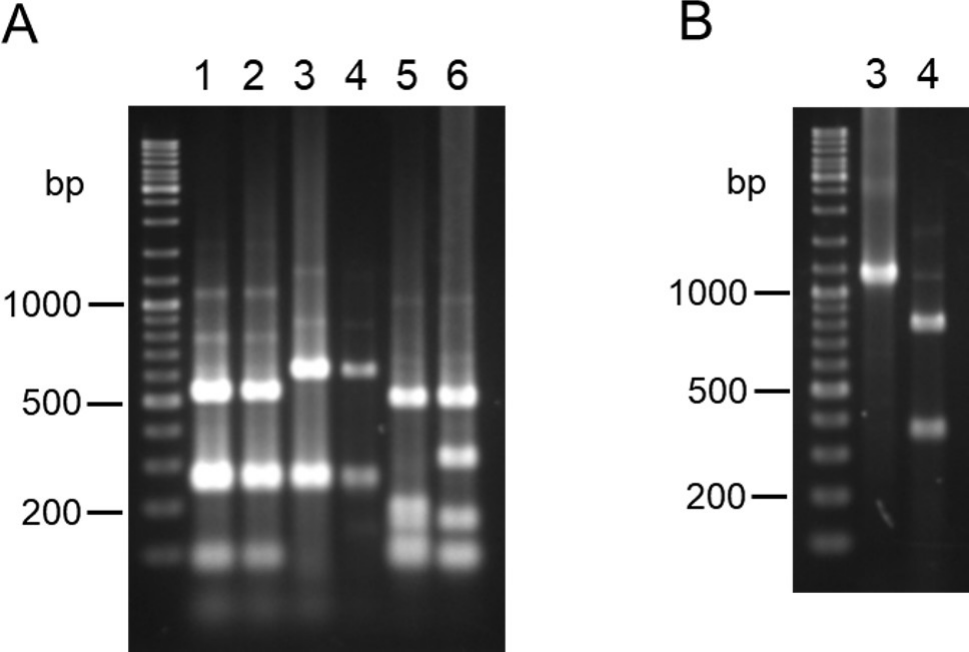
PCR-RFLP analyses of *mpi* gene fragments from 6 *Leishmania* species in Ecuador. PCR amplification was performed with leishmanial *mpi* gene-specific primers, and PCR products were digested with (A) *Hae*III and (B) *Hpa*I, and resulting restriction fragment patterns were analyzed by agarose gel electrophoresis. 1. *L*. *(V*.*) guyanensis*, 2. *L*. *(V*.*) panamensis*, 3. *L*. *(V*.*) braziliensis*, 4. *L*. *(V*.*) naiffi*, 5. *L*. *(L*.*) major*-like, 6. *L*. *(L*.*) mexicana*.

*Leishmania cyt* b, *hsp70*, *mpi* and *6pgd* partial gene sequences were amplified from 27 strains of 6 species isolated in Ecuador. Sequences of these fragments showed high degrees of homology (88–100%, 82–100%, 83–100% and 94–100% in *cyt* b, *mpi*, *6pgd* and *hsp70* genes, respectively) with corresponding leishmanial genes registered in GenBank. The restriction enzyme mapping was performed *in silico* to see if species-specific enzyme sites could be found in *cyt* b, *mpi*, *6pgd* and *hsp70* gene fragments obtained in this study. Species-specific RFLP patterns could not be obtained for the *cyt* b gene because of intraspecies genetic variations through the sequences. On the *hsp70* gene, restriction enzymes to differentiate *Leishmania* species were found; however, RFLP patterns including several smaller fragments (< 300 bp) were similar among species. Therefore, it seems difficult to identify the species based on RFLP patterns of *hsp70* using agarose gel electrophoresis in some cases because of the resolution. On the other hand, restriction enzyme sites that can differentiate *Leishmania* species in Ecuador were identified in *mpi* and *6pgd* genes, except for two very closely-related species, *L*. *(V*.*) guyanensis* and *L*. *(V*.*) panamensis*. Different RFLP patterns were obtained in *L*. *(V*.*) guyanensis/L*. *(V*.*) panamensis*, *L*. *(V*.*) braziliensis/L*. *(V*.*) naiffi*, *L*. *(L*.*) major*-like and *L*. *(L*.*) mexicana* for digested *mpi* gene fragments with a restriction enzyme *Hae*III (Fig 1A). Although an RFLP polymorphism was observed in one (strain PT27) of seven *L*. *(L*.*) mexicana* strains, it did not affect species identification (Table 3). *L*. *(V*.*) braziliensis* and *L*. *(V*.*) naiffi*, showing the same RFLP patterns as *Hae*III digestion, were differentiated by *Hpa*I digestion (Table 3, Fig 1B). Although *L*. *(V*.*) lainsoni*, a recently reported species in the Ecuadorian Amazon [20], showed the same RFLP patterns as *L*. *(V*.*) guyanensis/L*. *(V*.*) panamensis* when digested with *Hae*III and *Hpa*I, *Bst*XI-digestion successfully differentiated it from *L*. *(V*.*) guyanensis/L*. *(V*.*) panamensis*, as reported in Peruvian strains (S2 Fig) [28].

**Table 3:** Fragment size of leishmanial *mpi* and *6pgd* genes generated by digestion with selected restriction enzymes.

Leishmania*	**strain**	*mpi*		*6pgd*	
		*Hae*III	*Hpa*I	*Bsp*1286I	*Hin*fI
*L*.*guy*	EC4	21, 87, 246, 259, 517	1130	90, 1182	107, 155, 200, 307, 503
*L*.*guy*	EC6	21, 87, 246, 259, 517	1130	90, 1182	107, 155, 200, 307, 503
*L*.*guy*	EC7	21, 87, 246, 259, 517	1130	90, 1182	107, 155, 200, 307, 503
*L*.*guy*	EC8	21, 87, 246, 259, 517	1130	90, 1182	107, 155, 200, 307, 503
*L*.*guy*	EC9	21, 87, 246, 259, 517	1130	90, 1182	107, 155, 200, 307, 503
*L*.*guy*	EC11	21, 87, 246, 259, 517	1130	90, 1182	107, 155, 200, 307, 503
*L*.*guy*	EC12	21, 87, 246, 259, 517	1130	90, 1182	107, 155, 200, 307, 503
*L*.*guy*	XPEA1	21, 87, 246, 259, 517	1130	90, 1182	107, 155, 200, 307, 503
*L*.*guy*	LM3	21, 87, 246, 259, 517	1130	90, 1182	107, 155, 200, 307, 503
*L*.*pan*	G05	21, 87, 246, 259, 517	1130	90, 1182	107, 155, 200, 307, 503
*L*.*pan*	G06	21, 87, 246, 259, 517	1130	90, 1182	107, 155, 200, 307, 503
*L*.*pan*	G07	21, 87, 246, 259, 517	1130	90, 1182	107, 155, 200, 307, 503
*L*.*pan*	INH23	21, 87, 246, 259, 517	1130	90, 1182	107, 155, 200, 307, 503
*L*.*bra*	Ppa20	21, 246, 259, 604	1130	58, 90, 197, 927	107, 155, 200, 307, 503
*L*.*bra*	LASU22	21, 246, 259, 604	1130	58, 90, 197, 927	6, 107, 149, 200, 307, 503
*L*.*nai*	07tor	21, 246, 259, 604	352, 778	58, 90, 1124	155, 200, 414, 503
*L*.*nai*	13tor1	21, 246, 259, 604	352, 778	58, 90, 1124	155, 200, 414, 503
*L*.*nai*	13tor2	21, 246, 259, 604	352, 778	58, 90, 1124	155, 200, 414, 503
*L*.*maj*	G09	81, 95, 116, 158, 187, 493	1130	529, 743	83, 169, 200, 406, 414
*L*.*maj*	PT115	81, 95, 116, 158, 187, 493	1130	529, 743	83, 169, 200, 406, 414
*L*.*mex*	PT23	81, 91, 162, 303, 493	1130	392, 880	32, 83, 200, 382, 575
*L*.*mex*	PT27	81, 253, 303, 493	1130	392, 880	32, 83, 200, 382, 575
*L*.*mex*	PT29	81, 91, 162, 303, 493	1130	392, 880	32, 83, 200, 382, 575
*L*.*mex*	PT103	81, 91, 162, 303, 493	1130	392, 880	32, 83, 200, 382, 575
*L*.*mex*	HU3	81, 91, 162, 303, 493	1130	392, 880	32, 83, 200, 382, 575
*L*.*mex*	HU4	81, 91, 162, 303, 493	1130	392, 880	32, 83, 200, 382, 575
*L*.*mex*	HU6	81, 91, 162, 303, 493	1130	392, 880	32, 83, 200, 382, 575

*L.*guy*: *L*. *(V*.*) guyanensis*, *L*.*pan*: *L*. *(V*.*) panamensis*, *L*.*bra*: *L*. *(V*.*) braziliensis*, *L*.*nai*: *L*. *(V*.*) naiffi*, *L*.*maj*: *L*. *(L*.*) major*-like, *L*.*mex*: *L*. *(L*.*) mexicana*

The nucleotide sequence data reported in this paper will appear in the DDBJ, EMBL and GenBank databases under the accession numbers LC468908-LC468956.

**Fig 2:**
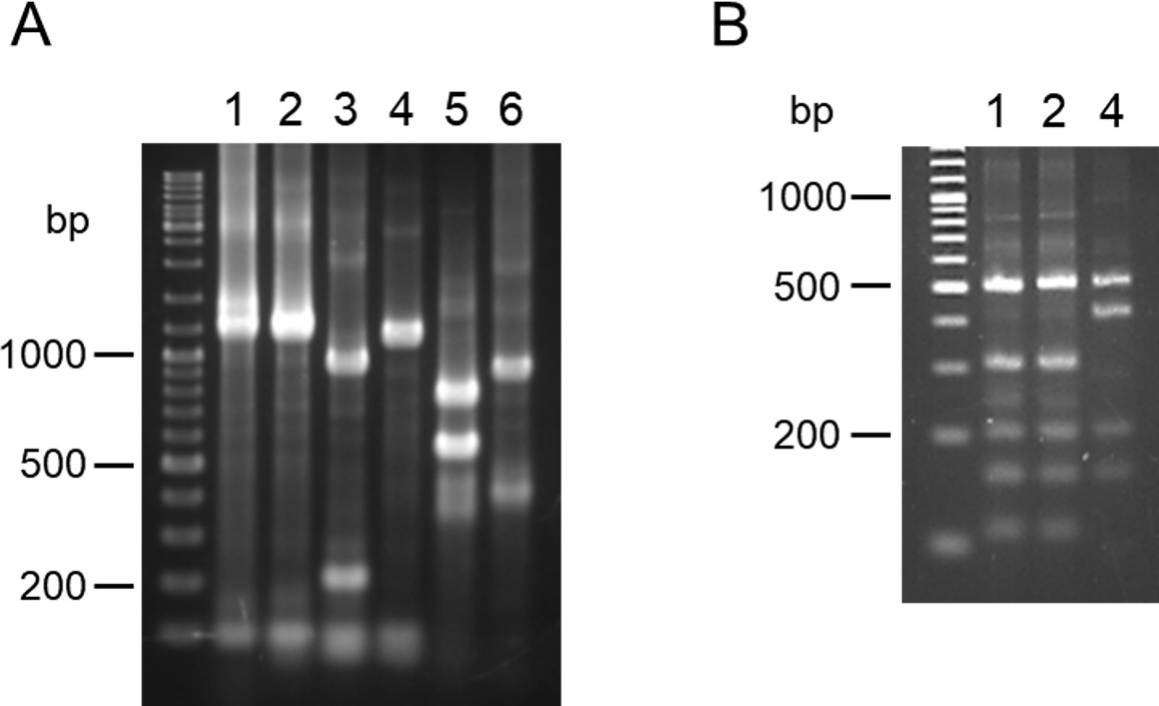
PCR-RFLP analyses of *6pgd* gene fragments from 6 *Leishmania* species in Ecuador. PCR amplification was performed with leishmanial *6pgd* gene-specific primers, and PCR products were digested with (A) *Bsp*1286I and (B) *Hin*fI, and resulting restriction fragment patterns were analyzed by agarose gel electrophoresis. 1. *L*. *(V*.*) guyanensis*, 2. *L*. *(V*.*) panamensis*, 3. *L*. *(V*.*) braziliensis*, 4. *L*. *(V*.*) naiffi*, 5. *L*. *(L*.*) major*-like, 6. *L*. *(L*.*) mexicana*.

Digestion of the *6pgd* gene with *Bsp*1286I resulted in distinct gene fragment patterns of *L*. *(V*.*) guyanensis/L*. *(V*.*) panamensis*, *L*. *(V*.*) braziliensis*, *L*. *(V*.*) naiffi*, *L*. *(L*.*) major*-like and *L*. *(L*.*) mexicana*; however, the patterns between *L*. *(V*.*) guyanensis/L*. *(V*.*) panamensis* and *L*. *(V*.*) naiffi* were similar and difficult to discriminate because of only about a 50 bp difference in a fragment of approximately 1 kbp (Fig 2A). The two species were successfully differentiated by digesting with *Hin*fI (Fig 2B).

**Fig 3:**
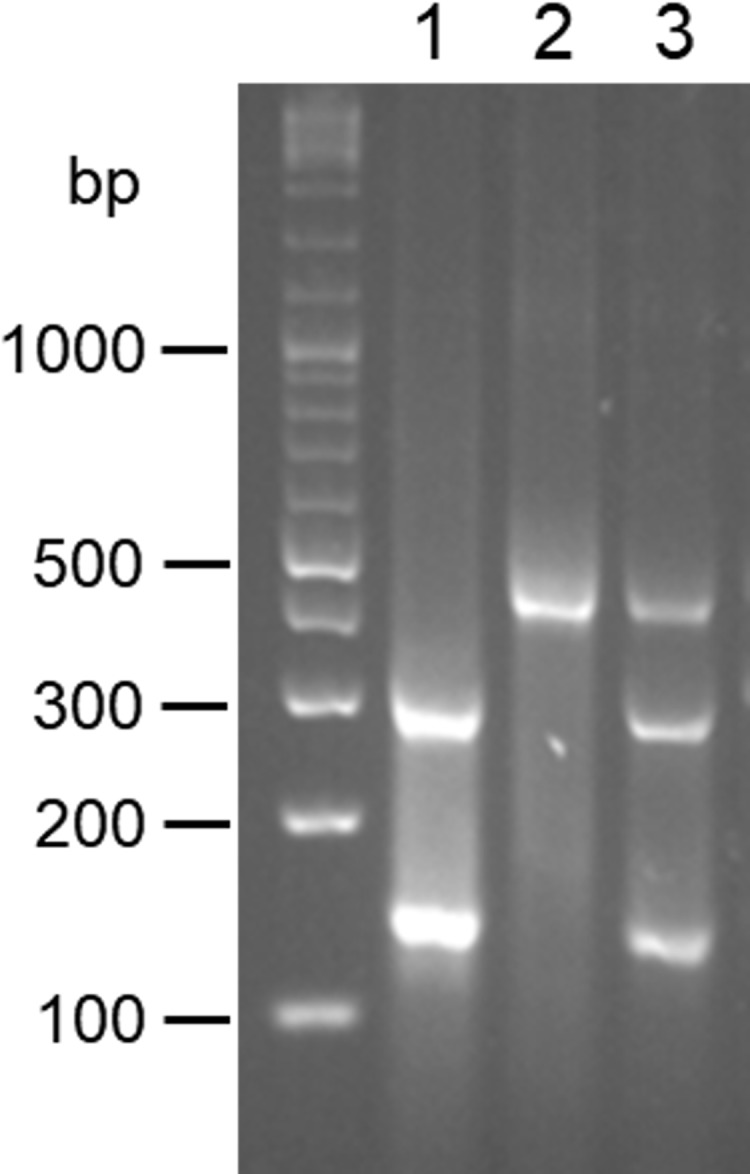
Differentiation between *L*. *(V*.*) guyanensis* and *L*. *(V*.*) panamensis* by PCR-RFLP of the *hsp*70 gene fragment. PCR amplification was performed with *hsp70* gene-specific primers and the PCR products were digested with *Bcc*I. 1. *L*. *(V*.*) guyanensis*, 2. *L*. *(V*.*) panamensis*, 3. a hybrid of *L*. *(V*.*) guyanensis* and *L*. *(V*.*) panamensis*.

Although *L*. *(V*.*) guyanensis* and *L*. *(V*.*) panamensis* were not discriminated by PCR-RFLP of *mpi* and *6pgd* genes, PCR-RFLP of the *hsp70* gene with a restriction enzyme, *Bcc*I, successfully differentiated the two species as reported previously (Fig 3) [27].

### Identification of *Leishmania* species in clinical samples by PCR-RFLP

**Fig 4:**
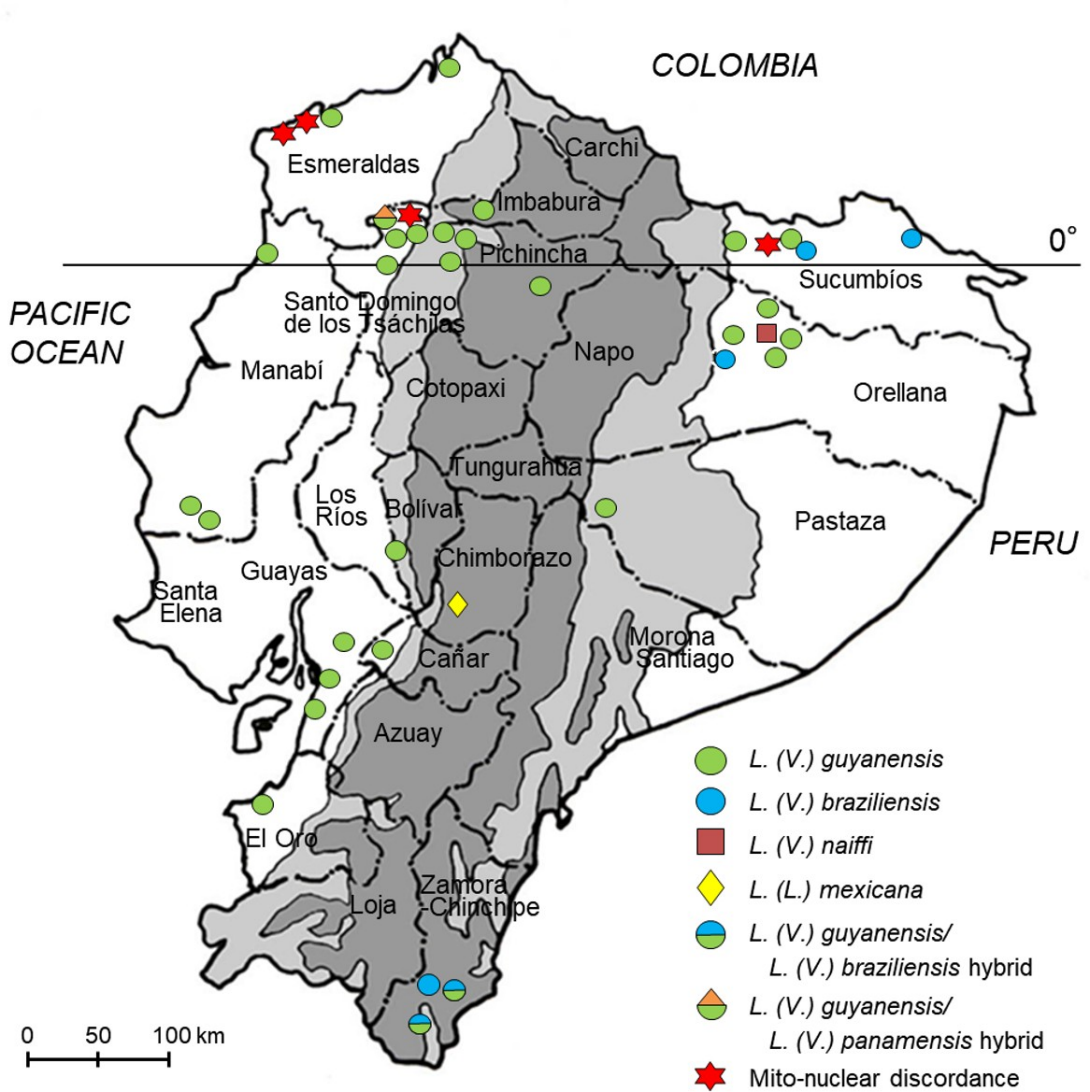
Geographic distribution of *Leishmania* species in Ecuador identified by PCR-RFLP analyses targeting multiple nuclear genes. The dark gray areas show the Andean plateau (>1,000 m altitude), and the light gray areas show highland jungle or Andean slopes (400–1,000 m elevation). (Adapted from a map available at http://english.freemap.jp/).

**Fig 5:**
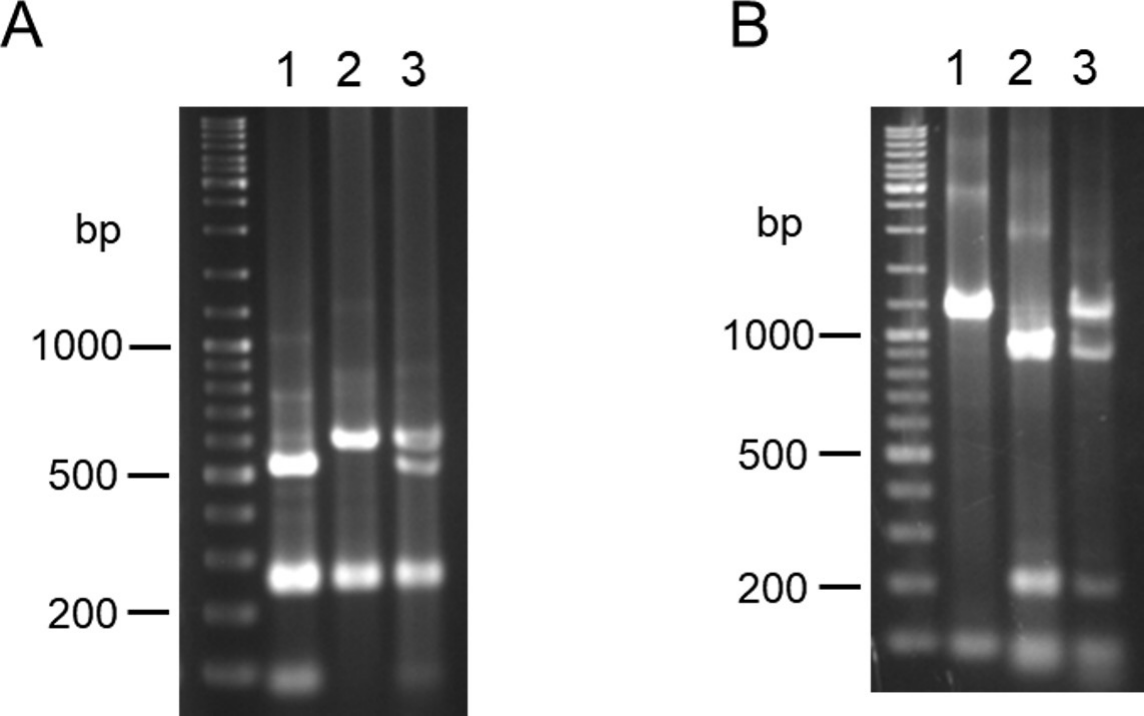
Differentiation between *L*. *(V*.*) guyanensis* and *L*. *(V*.*) braziliensis* by PCR-RFLP of *mpi* and *6pgd* gene fragments. A, B. PCR amplification was performed with *mpi* gene- or *6pgd* gene-specific primers and the PCR products were digested with *Hae*III (A) or *Bsp*1286I (B), respectively. 1. *L*. *(V*.*) guyanensis*, 2. *L*. *(V*.*) braziliensis*, 3. a hybrid of *L*. *(V*.*) guyanensis* and *L*. *(V*.*) braziliensis*.

PCR-RFLP analyses of *mpi* gene with restriction enzymes, *Hae*III and *Hpa*I, and *6pgd* gene with *Bsp*1286I and *Hin*fI were applied to 92 clinical samples collected at 33 sites in 14 provinces of Ecuador. PCR-RFLP analysis of the *hsp70* gene with a restriction enzyme, *Bcc*I, was used for differentiation between *L*. *(V*.*) guyanensis* and *L*. *(V*.*) panamensis*. The results obtained by PCR-RFLP analyses were compared with those obtained by the *cyt* b gene sequence analysis. The results of the species identification obtained by the two nuclear genes always agreed with each other. The identification by PCR-RFLP analyses completely matched with that obtained by the *cyt* b gene sequence analysis in all of *L*. *(V*.*) naiffi* (2 samples) and *L*. *(L*.*) mexicana* (3 samples) (Table 4). Of the 73 samples identified as *L*. *(V*.*) guyanensis* by *cyt* b gene analysis, 72 samples were identified as *L*. *(V*.*) guyanensis* by PCR-RFLP analyses, whereas one sample from a Pacific coast area showed a hybrid pattern of *L*. *(V*.*) guyanensis* and *L*. *(V*.*) panamensis* based on the PCR-RFLP of the *hsp70* gene (Figs 3 and 4). The sequence of the *hsp70* gene fragment was analyzed by direct sequencing, and a single nucleotide polymorphism was confirmed, showing “C” in *L*. *(V*.*) guyanensis* but “T” in *L*. *(V*.*) panamensis*, whereas a sample having a hybrid RFLP pattern had both “C” and “T” peaks at the corresponding position (S3 Fig), indicating the presence of a hybrid strain of *L*. *(V*.*) guyanensis* and *L*. *(V*.*) panamensis* in Ecuador. On the other hand, of the 14 samples identified as *L*. *(V*.*) braziliensis* by *cyt* b gene analysis, only 6 samples were identified as *L*. *(V*.*) braziliensis* by RFLP analyses (Table 4). In the other 8 samples identified as *L*. *(V*.*) braziliensis* by the *cyt* b gene analysis, three samples showed hybrid patterns in PCR-RFLP analyses of both the *mpi* and *6pgd* genes (Fig 5A and 5B). The sequences of *mpi* and *6pgd* gene fragments were analyzed by direct sequencing, and a single nucleotide polymorphism was confirmed, showing “C” in *L*. *(V*.*) guyanensis* but “T” in *L*. *(V*.*) braziliensis* of the *mpi* gene, and “T” in *L*. *(V*.*) guyanensis* but “C” in *L*. *(V*.*) braziliensis* of the *6pgd* gene. On the other hand, the *mpi* and *6pgd* genes from the three samples with hybrid RFLP patterns had both “C” and “T” peaks at the corresponding position (S4 Fig). From these results, the parasite species of these three samples were identified as a hybrid of *L*. *(V*.*) braziliensis* and *L*. *(V*.*) guyanensis* (Table 4, Fig 4). In the remaining 5 samples identified as *L*. *(V*.*) braziliensis* by sequence analysis of the *cyt* b gene, PCR-RFLP analyses showed that one sample from a Pacific coast area was *L*. *(V*.*) guyanensis*, three samples from the northern Pacific coast and Amazonian areas were *L*. *(V*.*) panamensis*, and one sample from a northern Pacific coast area had a hybrid pattern of *L*. *(V*.*) guyanensis* and *L*. *(V*.*) panamensis* (Table 4, Fig 4). The sequence analyses of *mpi*, *6pgd*, and *hsp70* gene fragments corresponded to PCR-RFLP analyses, indicating the presence of a mismatch between kinetoplast and nuclear genes, known as mito-nuclear discordance, in *Leishmania* distributing in Ecuador (Table 4, Fig 4). To further confirm the mito-nuclear discordance, partial sequences of the COII-ND1 region were analyzed as another target of kinetoplast genes in samples showing a mismatch between kinetoplast *cyt* b gene and nuclear *mpi*, *6pgd* and *hsp70* genes. The sequences were compared to each two corresponding sequences obtained from *L*. *(V*.*) braziliensis* and *L*. *(V*.*) guyanensis* in this study since this region has not been well-analyzed in subgenus *Viannia* species. The sequences from parasites with mito-nuclear discordance showed 98.9–99.1% and 98.5–98.9% identities with those of *L*. *(V*.*) braziliensis* and *L*. *(V*.*) guyanensis*, respectively (accession numbers: LC475135-LC475142). When partial COII gene sequences in the obtained COII-ND1 region sequences were analyzed on the GenBank database, the sequences from parasites with mito-nuclear discordance showed 99.5% and 98.9% identities with those of *L*. *(V*.*) braziliensis* and *L*. *(V*.*) guyanensis*, respectively. This result strongly suggested that the kinetoplast genes of these parasites originated from *L*. *(V*.*) braziliensis*, corresponding to the result of cyt b gene analysis.

**Table 4:** Comparison of *Leishmania* species identification in Ecuador between *cyt* b sequence analysis and PCR-RFLP analyses of nuclear DNAs.

**Target gene** **(analysis)**	Identification* (numbers)			
*cyt* b (cloning and sequencing)	L.g (73)	L.b (14)	L.n (2)	L.mex (3)
*mpi*, *6pgd*, and *hsp70* (PCR-RFLP)	L.g (72) L.g/L.p (1)	L.b (6) L.g/L.b (3) L.g^#^ (1) L.p^#^ (3) L.g/L.p^#^ (1)	L.n (2)	L.mex (3)

*L.g: *L*. *(V*.*) guyanensis*, L.p: *L*. *(V*.*) panamensis*, L.b: *L*. *(V*.*) braziliensis*, L.n: *L*. *(V*.*) naiffi*, L.mex: *L*. *(L*.*) mexicana*, L.g/L.p: a hybrid of *L*. *(V*.*) guyanensis* and *L*. *(V*.*) panamensis*, L.g/L.b: a hybrid of *L*. *(V*.*) guyanensis* and *L*. *(V*.*) braziliensis*

^#^mito-nuclear discordance

## Discussion

In the present study, PCR-RFLP analyses were employed for the identification of *Leishmania* species distributing in Ecuador in order to develop a simple and practical way for species identification independent of expensive equipment such as a genetic analyzer. As a result, *mpi* and *6pgd* genes, for which encoding enzymes have been widely used as the gold standard of species identification, were identified as suitable targets for this purpose in the tested samples. The results obtained by the PCR-RFLP analyses of multiple nuclear targets were compared to those of *cyt* b gene sequence analysis [7, 8, 29–36]. Although most results corresponded between the two analyses, PCR-RFLP revealed distribution of hybrid and mito-nuclear discordant *Leishmania* strains, which could not be identified only by *cyt* b gene sequence analysis. The results indicated that *Leishmania* strains distributing in Ecuador are genetically more complex than previously thought.

PCR-RFLP analysis has been employed for species identification of *Leishmania* species, and its utility is widely accepted [34]. The rRNA internal transcribed spacer 1 (ITS-1) region and *hsp70* gene are mostly used as suitable target genes, of which the former is applied mainly in the Old World [6, 11, 12, 14, 17, 19, 27, 34, 37–41]. Although the *hsp70* gene is one of the most valuable genetic markers for PCR-RFLP-based species identification, intraspecific polymorphism of RFLP patterns and very similar RFLP profiles among species, which affect species identification, have been reported in some *Leishmania* species [42]. In this study, other nuclear genes, *mpi* and *6pgd* genes, for which encoding enzymes have been used for MLEE, were shown to be alternative useful targets for classification by PCR-RFLP analysis. Of these, the *mpi* gene was reported to be the only genetic marker that can distinguish two very closely-related species, *L*. *(V*.*) braziliensis* and *L*. *(V*.*) peruviana* [7, 43, 44]. In addition, a recent study demonstrated that PCR-RFLP of the shorter *mpi* gene fragment (approximately 500 bp) can differentiate 4 *Leishmania* species [*L*. *(V*.*)braziliensis*, *L*. *(V*.*) peruviana*, *L*. *(V*.*) guyanensis*, and *L*. *(V*.*) lainsoni*] and a hybrid of *L*. *(V*.*) braziliensis* and *L*. *(V*.*) peruviana* circulating in the Department of Huanuco, Peru [28]. In the present study, PCR-RFLP analyses of longer *mpi* and *6pgd* gene fragments (>1000bp) were successfully established and applied to 92 clinical samples in Ecuador. Although a polymorphic RFLP pattern, which does not affect the identification, was detected in the *mpi* of one *L*. *(L*.*) mexicana* strain, the variant RFLP pattern was not detected in the present clinical samples identified as *L*. *(L*.*) mexicana*. Further sample analyses from different areas and different countries will be important to confirm the utility of this analysis, although polymorphic RFLP profiles may be detectable in these genes. Since polymorphism was also reported in the *hsp70* gene of several *Leishmania* species [42], PCR-RFLP analyses of multiple target genes, rather than single nuclear or kinetoplast genes, will result in more accurate species identification and disclose more detailed genetic characteristics of the parasite.

Several samples showing hybrid RFLP patterns were identified as hybrid strains rather than mixed infection of different *Leishmania* species. It is due to the following reasons: 1) It is little or no chance to be infected by more than one parasite in a cutaneous lesion because the lesion is typically developed at the site bitten by a sand fly transmitting specific *Leishmania* species, 2) Even if mixed infection occurs, either parasite becomes dominant in the lesion, resulting in the presence of dominant allele by the genetic analysis. However, both alleles were comparably amplified as observed in the PCR-RFLP analysis, which is indicative of a putative hybrid strain. In addition, similar results were obtained on electrograms of the direct sequencing, showing comparable fluorescence intensities of polymorphic nucleotides derived from both species. 3) The presence of hybrid strain has been reported in the same area as described below [45]. Isolation of putative hybrid strains as a culture is necessary for further detailed characterization of these parasites.

Although multiple PCR-RFLP and *cyt* b sequence analyses showed corresponding results in most clinical samples, the present study revealed the distribution of several unexpected strains in Ecuador, including hybrid and mito-nuclear discordance strains. Since hybrid strains cannot be identified by the *cyt* b gene analysis after molecular cloning, this is another advantage of identifying parasite species by PCR-RFLP. Distribution of a hybrid strain of *L*. *(V*.*) guyanensis/panamensis* complex and *L*. *(V*.*) braziliensis* was reported in Zumba, a province of Zamora-Chinchipe in a southern part of Ecuador by using MLEE and random amplified polymorphic DNA (RAPD) [45]. The present study confirmed the presence of the hybrid strain in Zumba, and also in another area in the same province, Palanda. In addition, a hybrid of *L*. *(V*.*) guyanensis* and *L*. *(V*.*) panamensis* was detected in northern Pacific areas of Ecuador. This is the first report of the presence of a hybrid strain of *L*. *(V*.*) guyanensis* and *L*. *(V*.*) panamensis* in Ecuador. *L*. *(V*.*) guyanensis* and its closely related *L*. *(V*.*) panamensis* have been reported to be endemic in northern Pacific areas of Ecuador by MLEE; however, only *L*. *(V*.*) guyanensis* was identified in the same areas by *cyt* b gene analysis in recent studies [8, 21, 46]. The present study confirmed that *L*. *(V*.*) guyanensis* is dominantly present in these areas, suggesting that endemic species may change, or that there may be discordance between MLEE and genetic analysis. However, the identification of a hybrid of *L*. *(V*.*) guyanensis* and *L*. *(V*.*) panamensis* as a minor population suggests that parental *L*. *(V*.*) panamensis* may still be present in some of these areas. Another unexpected finding was identification of mito-nuclear discordant strains of *Leishmania* species in northern Pacific and Amazonian areas. Interestingly, mito-nuclear discordant strains were identified only in the species identified as *L*. *(V*.*) braziliensis* by *cyt* b gene analysis. This finding supports a recent study using *cyt* b gene analysis reporting increasing cases of *L*. *(V*.*) braziliensis* infection in Pacific coast areas when compared to previous studies using enzymatic MLEE analysis [8]. The hybrid strain of *L*. *(V*.*) braziliensis* and *L*. *(V*.*) peruviana* was suggested to increase disease severity when compared to parental species in an animal model [47]. Therefore, careful investigation is needed to clarify the presence of hybrid strains, including mito-nuclear discordance, and their effects on clinical courses. In addition, hybrid strains may increase the range of transmissible sand fly species if they have a potential to be transmitted by both vector species of parental parasites. Continuous vector research is important in these endemic areas, as well as parasitological and clinical studies. Further, basic parasitological research on how genetic exchange and mito-nuclear discordance occur among *Leishmania* species would be another interesting subject [48–51]. Mito-nuclear discordance is reported in various animals such as mammals, birds, reptiles, amphibians, fish and insects, and is inferred to result from various processes: 1) adaptive introgression of mitochondrial DNA, 2) demographic disparities, 3) sex-biased asymmetries, 4) hybrid zone movement, 5) an intracellular bacteria, *Wolbachia* infection in insects, and 6) human actions [52]. It provides deeper insights into the phylogenetic relationship, population structure, and evolutionary signature of these animals. Mito-nuclear discordance is also reported in helminth parasites: trematodes *Schistosoma turkestanicum* between populations [53], and cestodes *Taenia solium* between lineages [54], and between *T*. *saginata* and *T*. *asiatica* [55–57]. This is the first report of mito-nuclear discordance in protozoan parasites. Mito-nuclear discordance is speculated to be resulted from the similar process as hybridization of nuclear genes in protozoa. Further study is needed to disclose the mechanism of mito-nuclear discordance formation in protozoa. In addition, association of mito-nuclear discordance with the pathogenicity and vector competency of the parasites is important issues to be clarified. In this study, we established a novel PCR-RFLP-based genotyping approach to identify *Leishmania* species in Ecuador. Although the present PCR-RFLP analyses was shown to be practical for identification of *Leishmania* species in Ecuador, further study focusing on other *Leishmania* species and clinical samples from different countries will be needed to enhance the utility of this approach. PCR-RFLP analyses of clinical samples and subsequent comparison with kinetoplast *cyt* b sequence analysis revealed the distribution of genetically complex *Leishmania* strains having genetic characteristics of hybrid and mito-nuclear discordance. Although intraspecies genetic variation observed in the *cyt* b gene resulted in this gene as an unsuitable target for RFLP analysis, there is no doubt about the utility of *cyt* b gene sequence analysis for species identification and phylogenetic analysis since distinct interspecies genetic diversity of this gene overcomes the disadvantage of the intraspecies variation. However, the present study points to the importance of applying multiple target approaches as the combination of *cyt* b and the PCR-RFLP assays presented here, enhancing the reliability of species identification and characterization of genetic properties including hybrid and mito-nuclear discordance. Further studies are needed to reveal the parasitological characteristics of hybrid and mito-nuclear discordance, clinical outcomes caused by these parasites, and the range of vector species of these parasites. In addition, studies on mito-nuclear discordance in *Leishmania* and other protozoa may provide further insights into the mechanism of genetic exchanges of these parasites.

## Supporting information

S1 Fig Sample collection sites in Ecuador. The dark gray areas show the Andean plateau (>1,000 m altitude), and the light gray areas show highland jungle or Andean slopes (400–1,000 m elevation). 1. San Lorenzo, 2. Esmeraldas, and 3. Atacames, Province of Esmeraldas; 4. Pedernales, 5. Montalvo, and 6. Pedro Pablo Gomez, Province of Manabi; 7. Cielo Verde, Province of Imbabura; 8. Puerto Quito, 9. Pedro Vicente Maldonado, 10. Los Bancos, 11. Nanegalito, 12. Pachijal, and 13. Quinche, Province of Pichincha; 14. Valle Hermoso, Province of Santo Domingo; 15. Balsapamba, Province of Bolivar; 16. Chanchan, Province of Chimborazo; 17. La Troncal, Province of Cañar; 18. El Triunfo, 19. Naranjal, and 20. Balao, Province of Guayas; 21. Santa Rosa, Province of El Oro; 22. Cascales, 23. Lago Agrio, and 24. Palma Roja, Province of Scumbios; 25. Coca, 26. Shangrila, 27. La Joya de los Sachas, 28. Pompeya, 29. Union Milagrena, and 30. Loreto, Province of Orellana; 31. Puyo, Province of Pastaza; 32. Palanda, and 33. Zumba, Province of Zamora-Chinchipe. (Adapted from a map available at http://english.freemap.jp/)
(TIF)

S2 Fig PCR-RFLP analysis of *mpi* gene fragments from *L. (V.) guyanensis, L. (V.) panamensis*, and *L. (V.) lainsoni*. PCR amplification was performed with *mpi* gene-specific primers and the PCR products were digested with *Bst*XI. 1. *L*. *(V*.*) guyanensis*, 2. *L*. *(V*.*) panamensis*, 3. *L*. *(V*.*) lainsoni*.
(TIF)

S3 Fig Direct sequence analysis showing a species-specific polymorphic site of *Leishmania hsp70* gene fragments. (TIF)

S4 Fig Direct sequence analysis showing a species-specific polymorphic site of *Leishmania mpi* gene (A) or *6pgd* gene (B) fragments.
(TIF)
