# Supplementary figures and images for: PCR-RFLP analyses of Leishmania species causing cutaneous and mucocutaneous leishmaniasis revealed distribution of genetically complex strains with hybrid and mito-nuclear discordance in Ecuador
Source: PLoS Negl Trop Dis. 2019 May 6;13(5):e0007403. doi: 10.1371/journal.pntd.0007403 (PMC6522058; doi:10.1371/journal.pntd.0007403)

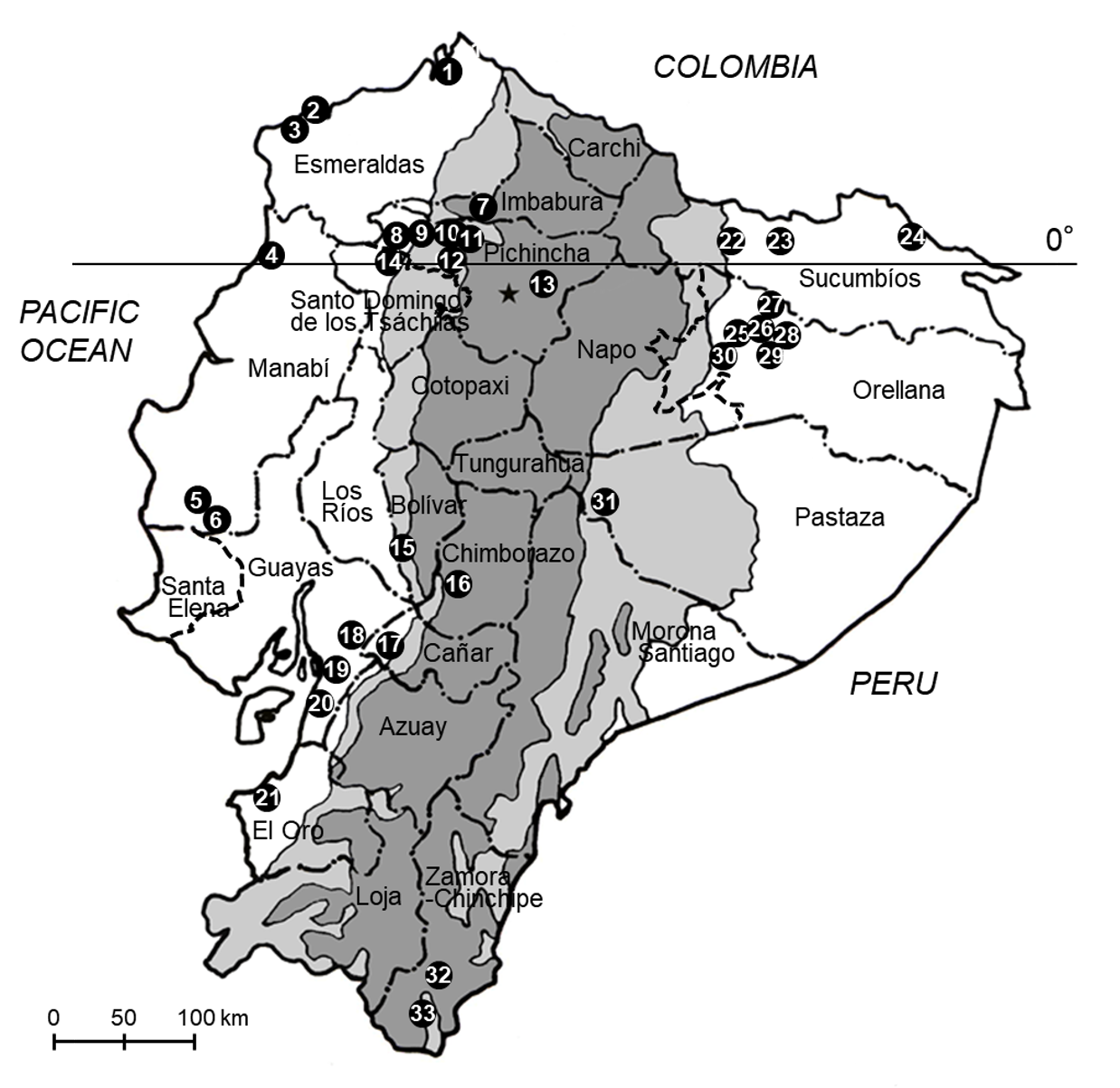

Supplement: S1 Fig — The dark gray areas show the Andean plateau (>1,000 m altitude), and the light gray areas show highland jungle or Andean slopes (400–1,000 m elevation). 1. San Lorenzo, 2. Esmeraldas, and 3. Atacames, Province of Esmeraldas; 4. Pedernales, 5. Montalvo, and 6. Pedro Pablo Gomez, Province of Manabi; 7. Cielo Verde, Province of Imbabura; 8. Puerto Quito, 9. Pedro Vicente Maldonado, 10. Los Bancos, 11. Nanegalito, 12. Pachijal, and 13. Quinche, Province of Pichincha; 14. Valle Hermoso, Province of Santo Domingo; 15. Balsapamba, Province of Bolivar; 16. Chanchan, Province of Chimborazo; 17. La Troncal, Province of Cañar; 18. El Triunfo, 19. Naranjal, and 20. Balao, Province of Guayas; 21. Santa Rosa, Province of El Oro; 22. Cascales, 23. Lago Agrio, and 24. Palma Roja, Province of Scumbios; 25. Coca, 26. Shangrila, 27. La Joya de los Sachas, 28. Pompeya, 29. Union Milagrena, and 30. Loreto, Province of Orellana; 31. Puyo, Province of Pastaza; 32. Palanda, and 33. Zumba, Province of Zamora-Chinchipe. (Adapted from a map available at http://english.freemap.jp/) (TIF) [file pntd.0007403.s001.tif]

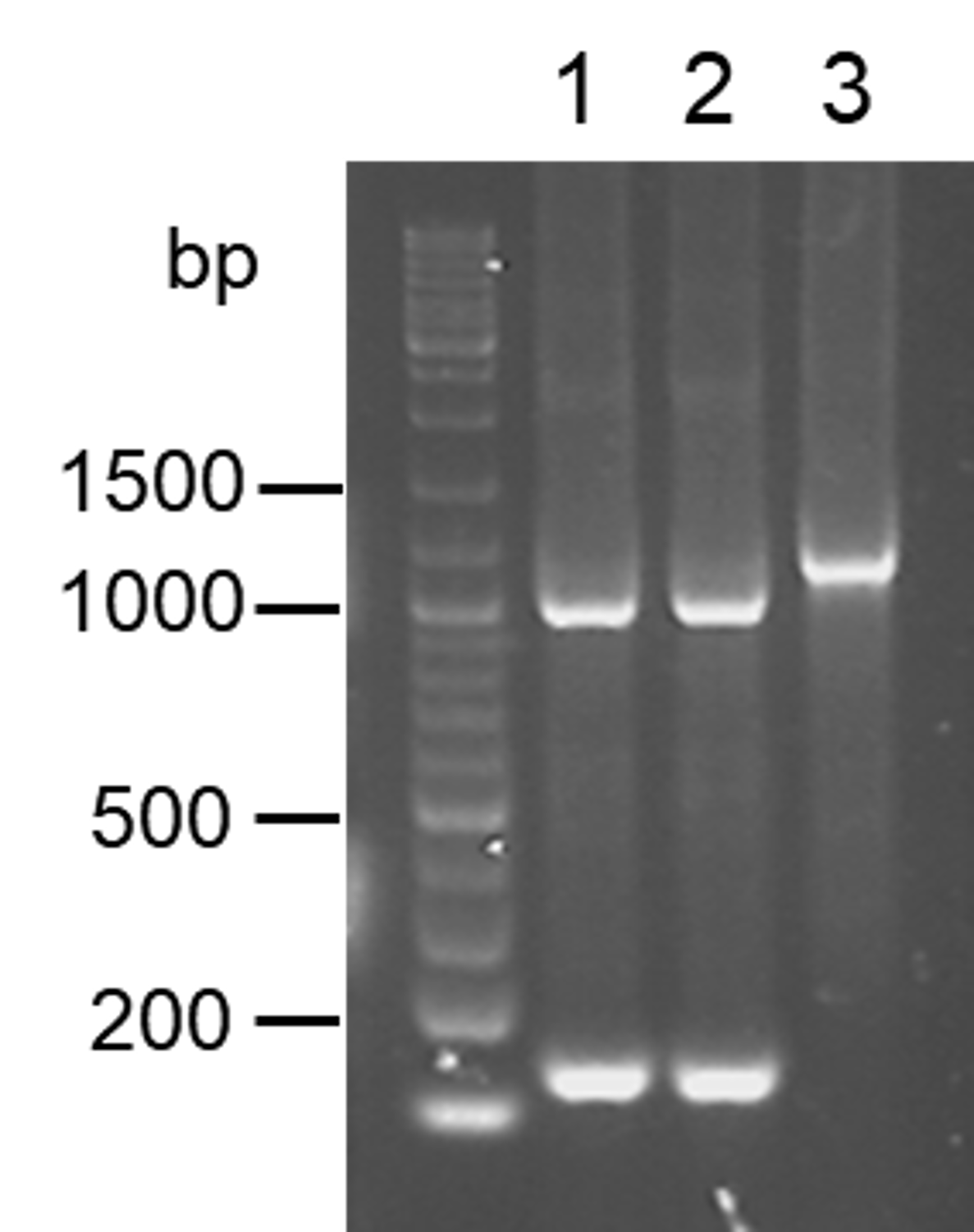

Supplement: S2 Fig — 1. L. (V.) guyanensis, 2. L. (V.) panamensis, 3. L. (V.) lainsoni. (TIF) [file pntd.0007403.s002.tif]

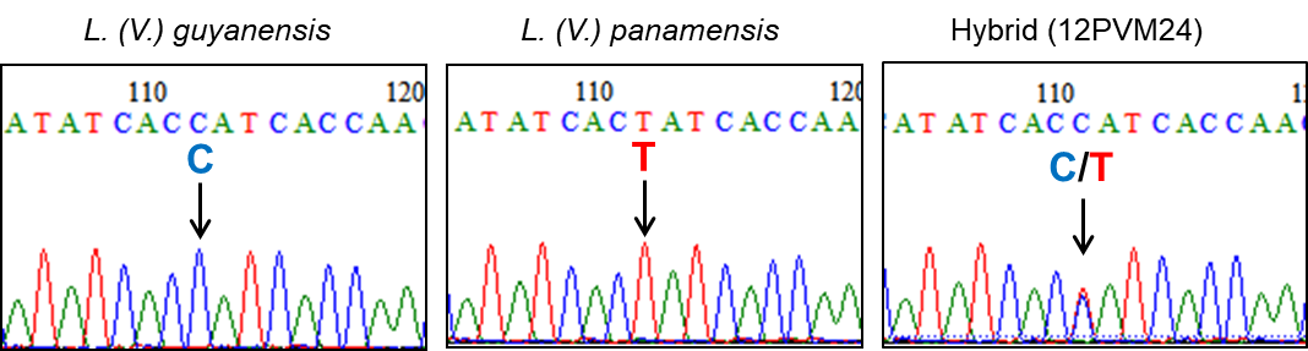

Supplement: S3 Fig — (TIF) [file pntd.0007403.s003.tif]

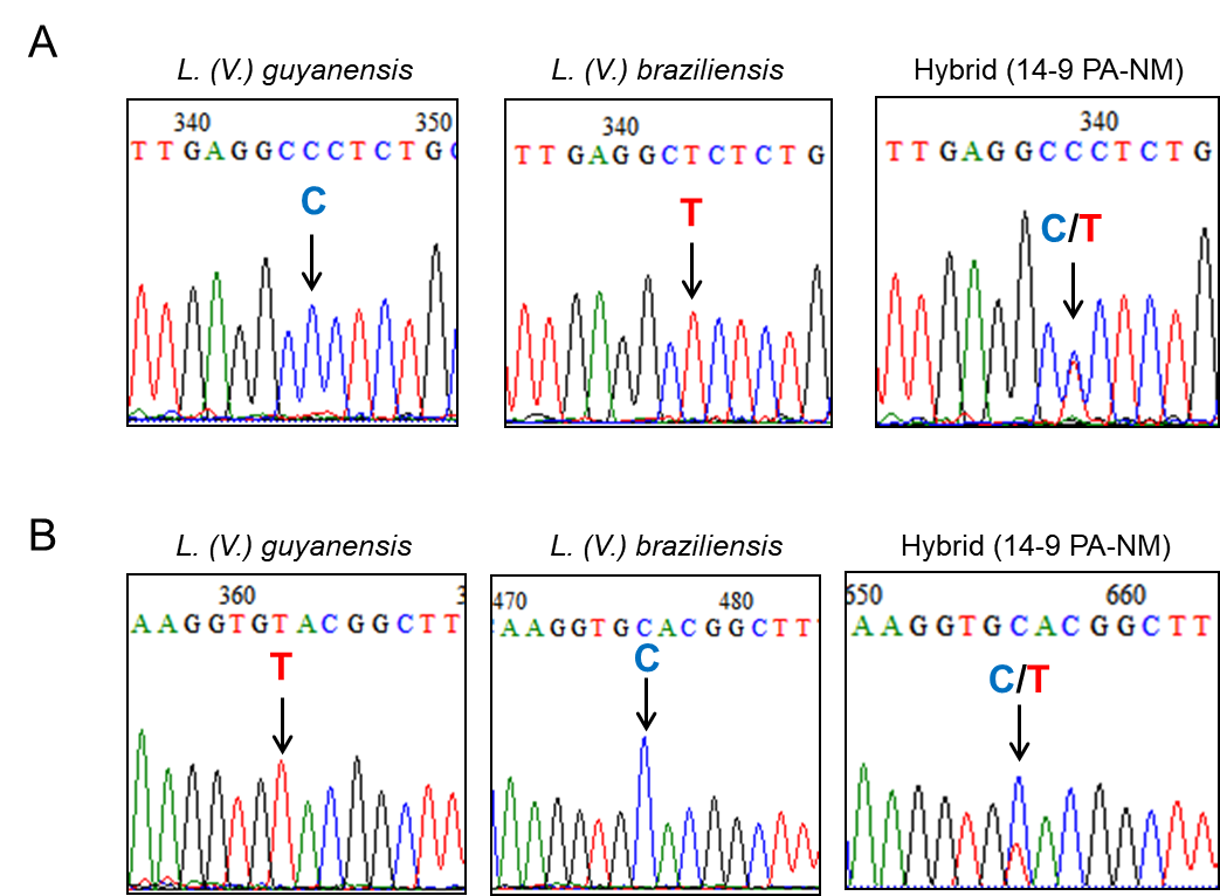

Supplement: S4 Fig — Direct sequence analysis showing a species-specific polymorphic site of Leishmania mpi gene (A) or 6pgd gene (B) fragments. (TIF) [file pntd.0007403.s004.tif]
